# Antibiotic Resistance and Virulence Traits in Vancomycin-Resistant Enterococci (VRE) and Extended-Spectrum β-Lactamase/AmpC-producing (ESBL/AmpC) *Enterobacteriaceae* from Humans and Pets

**DOI:** 10.3390/antibiotics9040152

**Published:** 2020-03-31

**Authors:** Ramona Iseppi, Alessandro Di Cerbo, Patrizia Messi, Carla Sabia

**Affiliations:** 1 Department of Life Sciences, University of Modena and Reggio Emilia, Via G. Campi 287, 41125 Modena, Italy; patrizia.messi@unimore.it (P.M.); carla.sabia@unimore.it (C.S.); 2 School of Biosciences and Veterinary Medicine, University of Camerino, Via Circonvallazione 93/95, 62024 Matelica, Italy

**Keywords:** virulence factors, VRE, ESBL, humans, dogs, cats

## Abstract

Background: We investigated the virulence factors, genes, antibiotic resistance patterns, and genotypes (VRE and ESBL/AmpC) production in Enterococci and *Enterobacteriaceae* strains isolated from fecal samples of humans, dogs, and cats. Methods: A total of 100 fecal samples from 50 humans, 25 dogs, and 25 cats were used in the study. MICs of nine antimicrobials were determined using the broth microdilution method. Polymerase chain reaction was used for the detection of genes responsible for antibiotic resistance (VRE and ESBL/AmpC) and virulence genes both in *Enterococcus* species, such as cytolysin (cylA, cylB, cylM), aggregation substance (agg), gelatinase (gelE), enterococcal surface protein (esp), cell wall adhesins (efaAfs and efaAfm), and in *Enterobacteriaceae*, such as cytolysin (hemolysin) and gelatinase production (afa, cdt, cnf1, hlyA, iutA, papC, sfa). Results: *Enterococcus faecium* was the most prevalent species in humans and cats, whereas *Enterococcus faecalis* was the species isolated in the remaining samples. A total of 200 *Enterobacteriaceae* strains were also detected, mainly from humans, and *Escherichia coli* was the most frequently isolated species in all types of samples. In the *Enterococcus* spp, the highest percentages of resistance for ampicillin, amoxicillin/clavulanate, erythromycin, tetracycline, ciprofloxacin, teicoplanin, and vancomycin were detected in cat isolates (41.6%, 52.8%, 38.9%, 23.6%, 62.5%, 20.8%, and 23.6% respectively), and in *E. coli*, a higher rate of resistance to cefotaxime and ceftazidime emerged in cat and dog samples, if compared with humans (75.4% and 66.0%, 80.0% and 71.4%, and 32.0% and 27.2%, respectively). Regarding the total number of enterococci, 5% and 3.4% of the strains were vancomycin and teicoplanin resistant, and the vancomycin resistance (van A) gene has been detected in all samples by PCR amplification. All the *Enterobacteriaceae* strains were confirmed as ESBL producers by PCR and sequencing, and the most frequent ESBL genes in *E. coli* strains from humans and pet samples were *bla*_CTX-M-1_ and *bla*_CTX-M-15_. Conclusions: Our results provide evidence that one or more virulence factors were present in both genera, underlining again the ability of pet strains to act as pathogens.

## 1. Introduction

Antimicrobial resistance (AMR) is a major health problem rapidly spreading across the world [1], and the interest of the scientific community in the presence and circulation of resistant organisms between pets and humans has also increased due to the important public health implications. *Enterococcus* spp. and *Enterobacteriaceae* represent important human and veterinary pathogens/opportunists and a significant burden for healthcare systems worldwide. The presence of these bacterial species in the gastrointestinal tract of both species plays a role in maintaining the normal digestive and immune functions of the hosts [2,3]. In addition, these bacteria species have also been found to participate in metabolic activities that save energy and absorbable nutrients as well as protect the colonized host against invasion by foreign microbes [4].

Although these microorganisms are commensal in the gut of most animals and common in environments contaminated by human and animal feces, they emerged as nosocomial and community-acquired pathogens for their ability to develop high-level resistance to antimicrobials [5]. Enterococci are common hospital-acquired pathogens, accounting for 9.6% of all healthcare-associated infections in European countries [6], but knowledge about their epidemiology is still unclear and the possible zoonotic spread of antimicrobial-resistant enterococci is controversial. Studies on the virulence factors of *Enterococcus* strains such as cytolysin, aggregation substance (AS), gelatinase, antibiotic susceptibility, and genetic heterogeneity showed differences among the strains according to their host origins [7,8,9].

The introduction of cefotaxime and, generally, the third-generation cephalosporins was a milestone in antimicrobial chemotherapy. As a result of selective pressure exerted by these new cephalosporins, resistance in enterobacterial species emerged a few years later [10]. Extended-spectrum-β-lactamase (ESBL) and plasmid mediated (p)AmpC-producing *Enterobacteriaceae* have been isolated from humans, different animal species, and environments worldswide. ESBL/AmpC producing *Enterobacteriaceae* in humans and animals have also been increasingly reported [11,12]. Despite many studies on ESBL/AmpC-producing bacteria from different sources, clear data, especially on routes of transmission, are still lacking and, consequently, the epidemiology of ESBL/pAmpCs is also poorly understood. One of the driving forces behind the increased resistance is the use of third and fourth generation cephalosporins in humans and animals [13]. The European Medicines Agency recommends the use of these antimicrobials only when there are no other alternative authorized antimicrobials for the respective target species and indications [14]. In companion animals, among others, the first-generation cephalosporin cephalexin and third-generation, long-acting cephalosporin cefovecin are commonly used and licensed in Europe. Several studies have reported the presence of ESBL/pAmpC-producing *Enterobacteriaceae* in clinical samples from companion animals [15,16,17]; however, knowledge about the intestinal carriage of ESBL/AmpCs in healthy companion animals is limited [18,19].

Given that companion animals have been suggested as potential reservoirs for antimicrobial-resistant bacteria [18,20], we studied the circulation of resistant bacteria in pets to obtain a deeper knowledge on the possible role in transmission to humans. For this purpose, we investigated virulence factors, genes, antibiotic resistance patterns, and genotypes (VRE and ESBL/AmpC) production in *Enterococcus* and *Enterobacteriaceae* strains isolated from fecal samples of humans, dogs, and cats.

## 2. Results

### 2.1. Isolation and Identification of Bacterial Strains

A total of 200 enterococcal isolates were recovered from 100 fecal samples of humans, dogs, and cats. The preliminary identification of the isolates to species level by biochemical tests was confirmed by PCR results in all the isolates. Table 1 shows the distribution of enterococcal species in samples of different origin.

*Enterococcus faecium* was the most prevalent species in humans, cats and dogs (80%, 72.2% and 50% respectively), and *Enterococcus faecalis* was the only species isolated in the remaining samples (1.7% in humans, 27.7% in cats and 50% in dogs). No difference in the percentage of recovery between the two enterococcal species has emerged in dog isolates. A total of 200 *Enterobacteriaceae* strains were also detected; 110 from humans, 55 from cats, and 35 from dogs (Table 1). *Escherichia coli* was found to be the most isolated species in all types of samples (93.6%, 96.3%, and 71.4% in humans, cats, and dogs, respectively), followed by *Citrobacter freundii* (3.8%, 36.3%, and 28.5% in humans, cats, and dogs, respectively). Lastly, three *Klebsiella pneumoniae* strains (2.7%) were recovered in human samples only.

### 2.2. Antimicrobial Susceptibility Test

Table 2 shows the levels of antimicrobial resistance detected in enterococcal isolates, according to isolated species.

The highest percentages of resistance for ampicillin, amoxicillin/clavulanate, erythromycin, tetracycline, ciprofloxacin, teicoplanin, and vancomycin were detected in cat isolates (41.6%, 52.8%, 38.9%, 23.6%, 62.5%, 20.8%, and 23.6% respectively), compared with human isolates (18.5%, 17.5%, 8.7%, 11.2%, 15%, 25%, and 1.2%, respectively); intermediate levels of resistance to these antibiotics were observed in dog isolates (18.7%, 12.5%, 25%, 6.2%, 6.2%, 4.2%, and 6.2%, respectively) (Table 2). With respect to streptomycin, a higher percentage of resistance in human isolates (8.7%) than in those of dogs (2.8%) and cats (0%) was observed. All the strains resulted sensitive to Tigecycline. Fisher’s exact test conducted on each antibiotic resistance percentages found in *E. faecium* and *E. faecalis* for each species revealed no statistical significance, confirming the almost absolute independendent occorrunce of the resistance percentages from the operator’s action. The only exceptions were represented by cat ampicillin (*p < 0.05) and amoxicillin/clavulanate (*p < 0.01) resistance percentages, possibly indicating a bias due to the operator.

Table 3 shows the percentage of antibiotic susceptibility pattern in *E. coli* and *C. freundii* strains, which are the two most frequently isolated *Enterobacteriaceae* species from samples of different origins, and in *K. pneumoniae* spp., which was recovered from human samples only.

All three species were sensitive to meropenem and imipenem. A high percentage of resistance, up to 100%, was in general observed for ampicillin and amoxicillin–clavulanic acid, except for *C. freundii* of cat origin (50%). A high rate of resistance to cefotaxime and ceftazidime was noticed in *C. freundii* and *K. pneumonia* strains of human origin and for cefotaxime in *C. freundii* isolated from cat. An intermediate level of resistance to cefotaxime was both observed in *C. freundii* from cat (50%) and dog isolates (50%), the latter also having a reduced sensitivity to ceftazidime. With regard to *E. coli*, a high percentage of resistance to cefotaxime and ceftazidime emerged in cat and dog samples, if compared with humans. Lastly, a higher rate of resistance to tetracycline was observed in *E. coli* of cat origin (52.8%), rather than in human and dog isolates (9.71% and 0%, respectively). It was interesting that *E. coli* and *C. freundii* from all species had multiresistance to ampicillin, amoxicillin/clavulanate, cefotaxime, ceftazidime, and cefepime, while only *K. pneumonie* from humans had multiresistance to the aforementioned antibiotics. As for cats and humans, an intriguing multiresistance to tetracycline and ciprofloxacin was observed in *E. coli*. A Chi-square test for trend conducted on each antibiotic resistance percentage found in *E. coli, C. freundii*, and *K. pneumoniae* of humans revealed a statistical significance only for cefotaxime (**p < 0.01) and ceftazidime (***p < 0.001), possibly indicating a bias due to the operator. Coversely, Fisher’s exact test conducted on each antibiotic resistance percentage found in *E. coli*, *C. freundii*, and *K. pneumoniae* of pets revealed no statistical significance, confirming the absolute independendent occorrunce of the resistance percentages from the operator’s action.

### 2.3. Detection of Van Genes

On the total number of isolates, 10% and 9% of the strains were vancomycin and teicoplanin resistant (Table 2 and Table 3). The highest rate of resistance was observed in both *E. faecium* and *E. faecalis* strains from cats (19.2% and 20.8% for teicoplanin and 21.1% and 25% for vancomycin, respectively). Resistance to the same antibiotics was also detected in dog samples, but this was mainly related to *E. faecium* species (8.3 and 12.5 for teicoplanin and vancomycin, respectively). With regard to human samples, one *E. faecium* isolate only was insensitive to teicoplanin. Lastly, vancomycin resistance genes (van A) have been detected in all samples by PCR amplification.

### 2.4. Detection and Sequencing of Extended Spectrum Lactamase and AmpC Genes

Almost all isolates carried at least one ESBL/AmpC gene (Table 4). All the strains, 83.6% isolated from humans, 88.6% from dogs, and 43.6% from cat, were confirmed as ESBL producers by PCR and sequencing. In particular, the ESBL-producing strains isolated from humans belonged to the following genes (species/number of isolates): *bla*_TEM-92_, *bla*_TEM-20_, and *bla*_TEM-52_ (*E. coli*/20, 8, and 10, respectively), *bla*_CTX-M-1_ (*E. coli*/18, *K. pneumoniae*/8), and *bla*_CTX-M-15_ (*E. coli*/30, *C. freundii*/2). Among cat and dog isolates, the *bla*_TEM-52_ and *bla*_CTX-M-1_ were carried by *E. coli*/2 and 5, *E. coli*/8 and *E. coli*/9 respectively. Lastly, *bla*_CTX-M-15_ was recovered in both cat and dog isolates (*E. coli*/11 and *C. freundii*/1 in cat isolates; *E. coli*/8 and *C. freundii*/2 in dog isolates), whereas *bla*_CTX-M-14_ was detected in cat isolates only (*E. coli*/2).

Non-ESBL gene *bla*_TEM-1_ was the most frequently identified β-lactamase gene among all the isolates. It was detected in 16.3% of the human isolates and in 56.3% and 40% of cat and dog isolates.

The AmpC gene *bla*_MIR-1_ was detected in two human isolates (*E. coli*), while two cat and one dog isolates *E. coli* harbored the gene *bla*_CMY-2_ (Table 4).

### 2.5. Detection of Virulence Factors in Enterococcus Species

The distribution of the virulence factors and genes in *Enterococcus* species by their origins are given in Table 5.

Gelatinase and gelE were detected in 31.2% and 33.7%, in 22.2% and 13.9%, and in 16.6% and 4.1% of human, cat, and dog isolates, respectively. With regard to AS, encoded by agg., it was detected in 11.2% and 10% of human isolates and in 2.7% and 0% of cat isolates, respectively. The lack of AS and agg. gene in dog isolates suggests a low virulence and a reduced capability of the strains to show virulence traits. The absence of cytolysin and its respective genes (cylA, cylB) was observed in cat and dog isolates, as opposed to human ones (6.2%), while the cylM gene was detected in 7.5%, 4.1%, and 2% of human, cat, and dog isolates, respectively. The esp gene was present but with low prevalence (12.5%) in human isolates only. Lastly, the efaAfs and efaAfm genes were found in 26.2%, 12.5%, and 16.6%, and in 15%, 11.1%, and 16.6% of human, cat, and dog isolates, respectively.

### 2.6. Detection of Virulence Factors in Enterobacteriaceae

The distribution of the virulence factors and genes in *Enterobacteriaceae* by their origins are given in Table 3B. Gelatinase and cytolysin were detected in *E. coli* species only, in 27.2% and 8.7%, in 30.2% and 3.7%, and in 32% and 0%, of human, cat, and dog isolates, respectively.

The detection of virulence factors using PCR revealed that 95 (47.5%) of all strains (*n* = 200) were positive for at least one of the virulence genes tested (except for the cdt gene); of these strains, 53 (56.7%) were humans, 19 (34.5%) were cats, and 24 (68.5%) were dogs. In the strains isolated from humans, afa, cnf1, hlyA, iutA, papC, and sfa were the most common virulence genes identified in *E. coli* (0.9%, 11.6%, 5.8%, 5.8%, 14.5%, and 9.7%, respectively), followed by papC in *C. freundii* (50%) and in *K. pneumoniae* (18.8%). In cat and dog strains, hlyA (2%, 3%), iutA (4%, 8%), papC (10%, 5%), and sfa (2% and 8%) were the most common virulence genes, present only in *E.coli*. Lastly, cdt genes have never been detected in any species (Table 6).

## 3. Discussion

This study investigated the antibiotic resistance and virulence traits in enterococci and *Enterobacteriaceae* isolated from faeces of humans, dogs, and cats.

It is interesting to underline the low prevalence of *E. faecalis*, the most frequent enterococcal species detected in human infections [21,22] and fecal isolates, compared with *E. faecium*. Nevertheless, *E. faecalis* was the predominant enterococcal species in our fecal dog samples, similar to other studies [23,24].

Enterococci are commensal bacteria that possess natural gene transfer mechanisms that are able to spread multiple resistances. Therefore, it becomes crucial to study and characterize the strains isolated from household animals [25]. The enterococci isolated from pets possessed the highest rate of resistance to teicoplanin and vancomycin, whereas only one *E. faecium* of human source was insensitive to teicoplanin. Simjee et al. suggested that vancomycin-resistant enterococci may be acquired by humans through contact with dogs [26], which is a hypothesis that is also supported by Leener et al., who reported vancomycin resistance rates in pets higher than ours [27]. The presence in pets of enterococcus isolates with other biological traits such as gelatinase, aggregation substance, cytolysin, and the presence of their respective virulence genes (gelE, aggesp, cylA, cylB, cylM, efaAfsand, efaAfm) emphasizes the pathogenicity of these strains. The incidence of cytolysin in our study was lower than that previously reported by some researchers [28,29]. In total, the gelE gene was detected in 19.5% of all isolates and was the most common factor, as reported in other studies [28,30,31]. The esp was the less frequently detected virulence gene in dog and cat isolates, compared to human ones, which is a result that is consistent with previous reports [32,33,34,35,36]. Lastly, a lower percentage of strains producing hemolysin, gelatinase and AS emerged, when compared with the genotypic characterization. This may be due to the presence of silent and undetected genes or to the detection of a single gene inside an operon, which was obtained by PCR analysis. The conflicting results of our study versus other investigations concerning the occurrence of virulence factors among isolates might also be due to differences in the ecological origin of strains or to the sensitivity of different detection methods.

With regard to the identification of the *Enterobacteriaceae* strains, *Escherichia coli* was found to be the most frequently isolated species from all types of samples, followed by *Citrobacter freundii*, whereas *Klebsiella pneumoniae* was recovered in human samples only (three strains). The antibiotic resistance patterns of our isolates showed a high percentage of resistance to ampicillin and amoxixillin–clavulanic acid, as well as a high rate of resistance to cephalosporins. A higher rate of resistance to tetracycline was observed in *E. coli* of cat origin compared with isolates of other sources. All the strains tested were sensitive to carbapenems.

In light of recent in vivo acquisitions, it might be reasonable to hypothesize a possible link between the occurrence of a high rate of resistance to tetracycline in humans and pets and the occurrence of tetracyclines in their sera [37,38,39,40]. Moreover, several reports demonstrated that even respecting withdrawal times in intensive farming, a huge amount of oxytetracycline could be recovered in the bone of the animals, in particular chickens [41], and then transferred to the pet food chain, which employs a chicken bone percentage ranging from 20% to 30% for kibble production [38,42,43,44]. Thus, the chronic intake of such food might account for the presence of tetracyclines’ resistance in pets but also in humans, where the presence of chicken bone residues has been observed in Vienna sausages (data not shown).

The most frequent ESBL genes in humans and pet samples were *bla*_CTX-M-1_ and *bla*_CTX-M-15,_ especially in *E.coli* strain, as reported by other authors [15,45,46]. In the past, *bla*_CTX-M-15_ was merely associated with strains of human origin [47], and CTX-M-1 was the major CTX-M sub-type in cattle, pigs, poultry, and companion animal isolates in Europe [45,48,49]. Actually, this close correspondence between CTX-M types and isolates of human or animal origin is no longer so obvious. An increasing number of studies identified blaCTX-M-1 and *bla*_CTX-M-15_ in both types of populations [15,50], and our results confirm this trend. The resistance genes reported in this study are similar to those found in both humans and pet samples as reported elsewhere [15,50]. The survey of fimA, sfa, cnf1, papC, iutA, hlyA, and cdt genes circulation yielded results similar to those obtained in other studies [51,52,53,54]. The results provide evidence that two virulence factors (papC and hlyA) were present in all *E. coli* isolates; in fact, the association of hlyA with papC and sfa that was observed in the present research indicated that when hlyA is present in the genome, these other genes are present as well. This may be explained by the presence of a direct chromosomal linkage among these operons within particular DNA units on the chromosome, which are termed pathogenicity islands (PAIs) and carry virulence-associated genes coding sfa, hlyA, and papC [55,56]. The prevalence of virulence genes (cnf, hly, and pap) in humans here reported (31.9%) was closely similar to the results from Usein et al. [57] on the frequency of these genes (35, 5%) in fecal *E. coli* isolated from healthy adult humans.

Various epidemiological studies suggest that animals can carry VRE in their intestinal microbiota and be the source of VRE infection in humans (according to a classical zoonotic cycle) [58,59,60]. In fact, these VRE strains of animal origin can determine the colonization of human gut expressing their pathogenicity by transferring their resistance genes to other human intestinal bacteria [61]. In our study, the highest percentages of resistance teicoplanin and vancomycin were detected only in cat isolates. Vancomycin is an antibiotic of last resort in the treatment of Gram-positive bacterial infections, including enterococcal infections. The emergence of vancomycin-resistant enterococcal strains and the risk of transmission of resistance genes to the susceptible bacteria pose a serious risk to public health.

## 4. Material and Methods

### 4.1. Study Design

A total of 100 fecal samples, including 50 humans, coming from subjects of variable age (12–77 years) and sex (women and men), 25 dogs, and 25 cats were used in the study.

Every owner (>18 years old) chosen for the study had only one dog or one cat. The samples were collected for one year (2017), and the human and dog/or cat participants had not been using antimicrobials for at least three months prior to the study. Bacterial strains were selected using a protocol for growth in selective media containing antibiotics. The protocol of this study was submitted to the Research Ethics Committee of HUAP/UFF (CAAE0146.0.258.000-09) and the Animal Ethics Committee of the NAL/UFF (NAL00126-09), and it was approved and certified by both committees.

### 4.2. Isolation and Identification of Bacterial Strains

Fecal samples on swabs from dogs/cats and humans were obtained and processed for testing.

After enrichment growth in Tryptic Soy Broth (TSB, bioMerieux, Florence, Italy) supplemented with 4 mg/L vancomycin and 2 mg/L cefotaxime for vancomycin-resistant enterococci (VRE) and *Enterobacteriaceae* ESBL/AmpC, respectively, samples were seeded onto Bile Esculin Azide agar plates (bioMerieux, Florence, Italy) for VRE isolation and onto MacConkey agar (bioMerieux, Florence, Italy) for *Enterobacteriaceae* isolation and incubated at 37 °C for 24 h. For both genera, two different colonies were isolated.

Colonies with typical enterococcal morphology were identified at the genus level by cultural characteristics, Gram staining, the catalase test, and the bile aesculin reaction. Species identification was confirmed by polymerase chain reaction (PCR) using primers and conditions for the different enterococcal species [62,63].

Additionally, colonies with typical *Enterobacteriaceae* morphology (four per sample) were selected and identified by classical biochemical methods (Gram staining, catalase, oxidase, indole, Methyl-Red-Voges-Proskauer, citrate, and urease), and by the API 20E system (bioMerieux, Florence, Italy).

The isolates were confirmed using Vitek-2 (bioMerieux, Florence, Italy).

### 4.3. Antimicrobial Susceptibility Test

Antibiotic susceptibility was determined by the broth microdilution method on Mueller Hinton broth (Scharlab, Milan, Italy) according to the Clinical Laboratory Standards Institute (CLSI) [64]. As for Enterococci, ampicillin, amoxicillin/clavulanate, streptomycin, erythromycin, tetracycline, ciprofloxacin, teicoplanin, vancomycin, and tigecycline were tested, while ampicillin, amoxicillin/clavulanate, ciprofloxacin, tetracycline, cefotaxime, ceftazidime, cefepime, imipenem, and meropenem were tested on Enterobacteriaceae.

The Enterococcus faecalis strain ATCC 29,212 and Escherichia coli ATCC 25,922 were included to validate the antimicrobial susceptibility results as recommended for quality control by the European Committee on Antimicrobial Susceptibility Testing (EUCAST) [65].

### 4.4. Detection of Van Genes

DNA was extracted from the samples using a standard heat lysis protocol [66] and two vancomycin resistance-associated genes, vanA and vanB, were detected by PCR using primers that were used in previous studies [62]. Then, amplicons were analyzed on 1% agarose Tris-Borate-EDTA (TBE) gel containing GelRed^®^ (Sigma, Milan, Italy). Following gel electrophoresis at 150 V for 3 to 5 h, the images were recorded.

### 4.5. Detection and Sequencing of Extended Spectrum Lactamase and AmpC Genes

Regarding the molecular tests, DNA was extracted using a standard heat lysis protocol [66]. For the detection of *bla*_TEM_, *bla*_SHV_, and *bla*_CTX-M_ genes, the multiplex-PCR described by Kim et al. was used [67]. Furthermore, primer sets described by Perez-Perez and Hanson [66] to detect AmpC products were used. PCR-positive amplicons were purified by the QIAquick PCR Purification Kit (Qiagen, Milan, Italy) and directly sequenced using amplification primers on the 3130 Genetic Analyzer (Applied Biosystems, Milan, Italy). Purification and sequencing were carried out by Genex CZ, s.r.o. Sequence alignment and analysis were performed online using the BLAST program of the National Center for Biotechnology Information (www.ncbi.nlm.nih.gov).

### 4.6. Detection of Virulence Factors Cytolysin, Gelatinase, and Aggregation Substance (AS) Production in Enterococcus Species

As reported by Gulhan et al., Brain Heart Infusion agar (bioMerieux, Florence, Italy), supplemented with 5% horse blood, was used for the detection of cytolysin activity, while gelatinase production was evaluated using Todd-Hewitt agar (bioMerieux, Florence, Italy), and the measurement of the AS of the enterococci was performed by clumping assay [51].

### 4.7. Detection of Virulence Genes in Enterococcus Species by PCR

PCR amplification was performed to detect the presence of genes involved in the expression of cytolysin (cylA, cylB, cylM), aggregation substance (agg), gelatinase (gelE), enterococcal surface protein (esp), and cell wall adhesins (efaAfs and efaAfm), using the primers described by Eaton and Gasson (2001) [68].

### 4.8. Detection of Virulence Factors Cytolysin (Hemolysin) and Gelatinase Production in Enterobacteriaceae Species

Cytolytic protein toxins (alpha hemolysin) produced by most hemolytic bacteria were assayed by culturing on 5% sheep blood agar at 37 °C for 24 h. Hemolysin production was detected by the presence of a complete heamolytic zone around the colony [69].

Gelatinase production was assayed by inoculating test organisms on Gelatin agar and incubated at 37 °C for 24 h; the culture was flooded with mercuric chloride solution; thus, the development of opacity in the medium and a zone of clearance around the bacterial colonies were considered positive factors for the presence of gelatinase [70].

### 4.9. PCR detection of Virulence Genes of Enterobacteriaceae Species

Virulence genes were also detected by PCR, using primers targeting 7 extraintestinal putative virulence factors, which included adhesion genes (afa, papC, and sfa), toxin genes (cdt, cnf1, and hlyA), and the aerobactin receptor gene (iutA) [52,71,72,73,74].

#### Statistical Analysis

A Chi-squared exact test was used to compare the prevalence of resistance to antimicrobials simultaneously assayed on *E. faecium* and *E. faecalis* of all species and on *E. coli* and *C. freundii* of pets. Coversely, Fisher’s exact test was used to compare the prevalence of resistance to antimicrobials simultaneously assayed on *E. coli*, *C. freundii*, and *K. pneumoniae* of humans. All statistical analyses were performed with GraphPad Prism 8 (GraphPad Soft- ware Inc., San Diego, CA, USA). *p < 0.05 was considered significant.

## 5. Conclusions

Pets usually live in close contact with their owners, and this suggests the hypothesis that these animals, besides livestock [17], chickens, and pigs [75], might become reservoirs of multiresistant strains, representing a source of infection and a pool of resistance genes trasferable by conjugation to human pathogens. More data, in particular on plasmids and host bacteria, are required to draw any conclusions on genetic relatedness and the possible transmission of these “superbugs” from pets to human and vice versa.

## Figures and Tables

**Table 1:** Number (%) of isolates of *Enterococcus* and *Enterobacteriaceae* from 100 fecal samples of humans, cats, and dogs.

Enterococcusspecies	Humans (*n* = 80)	Cats (72)	Dogs (48)
*E. faecium*	68 (80)	52 (72.2)	24 (50)
*E. faecalis*	12 (1.5)	20 (27.7)	24 (50)
***Enterobacteriaceae* species**	Humans (*n* = 110)	Cats (55)	Dogs (35)
*E. coli*	103 (93.6)	53 (96.3)	25 (71.4)
*C. freundii*	4 (3.8)	2 (36.3)	10 (28.5)
*K. pneumoniae*	3 (2.7)	0	0

**Table 2:** Percentages of antimicrobial resistance in enterococci isolated from fecal samples of humans, cats, and dogs.

Antibiotic	Humans			Cats			Dogs		
	*E. faecium* (*n* = 68)	*E. faecalis* (*n* = 12)	Total (*n* = 80)	*E. faecium* (*n* = 52)	*E. faecalis* (*n* = 24)	Total (*n* = 72)	*E. faecium* (*n* = 24)	*E. faecalis* (*n* = 24)	Total (*n* = 48)
Ampicillin	11.7	33.3	18.5	28.8	62.5	41.6	25	12.5	18.7
Amoxicillin/clavulanate	11.7	25	17.5	38.4	75	52.8	16.7	25	12.5
Streptomycin	5.8	25	8.7	1.9	4.1	2.8	0	0	0
Erythromycin	10.3	16.7	11.2	20	20.8	38.9	25	25	25
Tetracycline	13.2	25	15	33.3	33.3	23.6	12.5	0	6.2
Ciprofloxacin	25.5	25	25	41.7	41.7	62.5	12.5	0	6.2
Teicoplanin	1.4	0	1.2	19.2	20.8	20.8	8.3	0	4.2
Vancomycin	0	0	0	21.1	25	23.6	12.5	0	6.2
Tigecycline	0	0	0	0	0	0	0	0	0

**Table 3:** Percentages of antimicrobial resistance in *Enterobacteriaceae* isolated from fecal samples of humans, cats, and dogs.

Antibiotic	Humans			Cats			Dogs		
	*E. coli* (*n* = 103)	*C. freundii* (*n* = 4)	*K. pneumoniae* (*n* = 3)	*E. coli* (*n* = 53)	*C. freundii* (*n* = 2)	*K. pneumoniae* (*n* = 0)	*E. coli* (*n* = 35)	*C. freundii* (*n* = 10)	*K. pneumoniae* (*n* = 0)
Ampicillin	79.7	100	100	86.7	50	0	85.7	100	0
Amoxicillin/clavulanate	77.6	100	100	86.7	50	0	85.7	100	0
Cefotaxime	32	75	100	75.4	100	0	80	50	0
Ceftazidime	27.2	75	100	66	50	0	71.4	50	0
Cefepime	24.2	50	50	60.3	25	0	68.6	50	0
Tetracycline	9.7	0	0	52.8	0	0	0	0	0
Ciprofloxacin	19.4	0	0	28.3	0	0	0	0	0
Imipenem	0	0	0	0	0	0	0	0	0
Meropenem	0	0	0	0	0	0	0	0	0

**Table 4:** Distribution of β-lactamase genes (%) in *Enterobacteriaceae* isolates of human cat and dog isolates.

Source	Humans			Cats			Dogs		
**N°. of Isolate ^a^**	*E. coli* (*n* = 103)	*C. freundii* (*n* = 4)	*K. pneumoniae* (*n* = 3)	*E. coli* (*n* = 53)	*C. freundii* (*n* = 2)	*K. pneumoniae* (*n* = 0)	*E. coli* (*n* = 25)	*C. freundii* (*n* = 10)	*K. pneumoniae* (*n* = 0)
Penicillinases ^b^									
*bla*_TEM-1_	14.5 (15)	50 (2)	33.3 (1)	60 (30)	50 (1)	0	40 (10)	40 (4)	0
ESBL ^b^						0			0
*bla*_TEM-92_	19.4 (20)			0	0	0			0
*bla*_TEM-20_	3.8 (8)			0	0	0			0
*bla*_TEM-52_	9.7 (10)	0	0	3.7 (2)	0	0	20 (5)	0	0
*bla*_CTX-M-1_	17.4 (18)	0	66.7 (2)	22.5 (8)	0	0	36 (9)	0	0
*bla*_CTX-M-14_	0	0	0	0	0	0	8 (2)	0	0
*bla*_CTX-M-15_	29.1 (30)	50 (2)	0	20.7 (11)	50 (1)	0	32 (8)	60(6)	0
AmpC ^b^				0			0	0	
*bla*_MIR-1_	19.4 (2)			0			0	0	0
*bla_CMY-2_*	0	0	0	3.7 (2)			4 (1)	0	0

**^a^** Numerous isolates encoded for more than one β-lactamase gene. **^b^** % of β-lactamase genes (n°. of isolates).

**Table 5:** Distribution of virulence factors and genes among *E. faecalis* and *E. faecium* isolates: cytolysin (cylA, cylB, cylM), aggregation substance (agg), gelatinase (gelE), enterococcal surface protein (esp), and cell wall adhesins (efaAfs and efaAfm).

Virulence Factors/Genes	Human *n* = 80(%)	Cat *n* = 72 (%)	Dog *n* = 48 (%)	Total *n* = 200 (%)
Gelatinase	25 (31.2)	16 (22.2)	8 (16.6)	49 (24.5)
* AS	9 (11.2)	2 (2.7)	0	11 (5.5)
Cytolysin	5(6.2)	0	0	5 (2.5)
gelE	27 (33.7)	10 (13.9)	2 (4.1)	39 (19.5)
cylA	3 (3.7)	0	0	3 (1.5)
cylB	3 (3.7)	0	0	3 (1.5)
cylM	6 (7.5)	3 (4.1)	1 (2)	10 (5)
agg	8 (10)	3 (4.1)	0	11 (5.5)
esp	10 (12.5)	0	0	10 (20)
efaAfs	21 (26.2)	9 (12.5)	8 (16.6)	38 (19)
efaAfm	12 (15)	8 (11.1)	8 (16.6)	28 (14)

* aggregation substance.

**Table 6:** Frequency of virulence factors and genes (%) among faecal *E. coli*, *C. freundii*, and *K. pneumoniae* isolated from human, cat, and dog samples.

Virulence Factors/Genes	Humans			Cats			Dogs			Total *n* = 200 (%)
	*E. coli* (*n* = 103)	*C. freundii* (*n* = 4)	*K. pneumoniae* (*n* = 3)	*E. coli* (*n* = 53)	*C. freundii* (*n* = 2)	*K. pneumoniae* (*n* = 0)	*E. coli* (*n* = 35)	*C. freudii* (*n* = 10)	*K. pneumoniae* (*n* = 0)	
Gelatinase	25 (27.2)	0	0	16 (30.2)	0	0	8 (32)	0	0	49 (24.5)
Cytolysin	(8.7)	0	0	2 (3.7)	0	0	0	0	0	11 (5.5)
afa	1 (0.9)	0	0	0	0	0	0	0	0	1 (0.5)
cdt	0	0	0	0	0	0	0	0	0	0
cnf1	12 (11.6)	0	0	0	0	0	0		0	12 (6)
hlyA	6 (5.8)	0	0	2 (3.7)	0	0	3 (12)	0	0	11 (5.5)
iutA	6 (5.8)	0	0	4 (7.5)	0	0	8 (32)	0	0	18 (9)
papC	15 (14.5)	2 (50)	1 (33.3)	10 (18.8)	0	0	5 (20)	0	0	33 (16.5)
sfa	10 (9.7)	0	0	2 (3.7)	0	0	8 (32)	0	0	20 (10)
